# Exploratory Multivariate Analysis of Mediator Organization in Canine Platelet-Rich Gel Under NSAID Exposure

**DOI:** 10.3390/gels12030246

**Published:** 2026-03-14

**Authors:** Jorge U. Carmona, Julián Ospina, Catalina López

**Affiliations:** 1 Grupo de Investigación Terapia Regenerativa, Departamento de Salud Animal, Universidad de Caldas, Calle 65 No. 26-10, Manizales 170004, Colombia; 2 Grupo de Investigación Patología Clínica Veterinaria, Departamento de Salud Animal, Universidad de Caldas, Calle 65 No. 26-10, Manizales 170004, Colombia; julianospina55@gmail.com

**Keywords:** platelet-rich gel, platelet-rich plasma, chemically induced platelet lysate, non-steroidal anti-inflammatory drugs, PDGF-BB/TNF-α ratio, principal component analysis, linear mixed-effects model, canine model, biobased biomaterials, cytokine balance

## Abstract

Platelet-rich gel (PRG) is a fibrin-based biobased biomaterial generated by activating platelet-rich plasma (PRP), yet its biological characterization has commonly relied on univariate measurements of isolated mediators. This study aimed to define the multivariate biological organization of PRG and related hemocomponents (PRP, chemically induced platelet lysate (CIPL), and plasma) in a canine model under single exposure to non-steroidal anti-inflammatory drugs (NSAIDs). In a randomized crossover design (n = 6 dogs), hemocomponents were produced at baseline (0 h) and 6 h after administration of carprofen or firocoxib. Platelet and white blood cell (WBC) counts, growth factors (platelet-derived growth factor-BB (PDGF-BB) and transforming growth factor beta-1 (TGF-β1)), and cytokines (tumor necrosis factor alpha (TNF-α), interleukin-1 beta, and interleukin-10) were integrated using linear mixed-effects modeling, principal component analysis (PCA), and hierarchical clustering. PRG was derived from a leukocyte-poor PRP precursor with moderate platelet enrichment (~1.6-fold vs. whole blood) and a marked WBC reduction (~8–9-fold). In mixed-effects modeling, hemocomponent type significantly influenced the PDGF-BB:TNF-α log-ratio, with PRG (estimate −1.12; 95% CI −1.34 to −0.90) and plasma (−2.06; 95% CI −2.28 to −1.84) lower than PRP, while CIPL did not differ. Time and NSAID effects were not supported. PCA identified two orthogonal axes explaining 61.3% of total variance (PC1 = 43.7%, PC2 = 18.6%), separating a platelet/trophic dimension (log(PDGF-BB), log(TGF-β1), platelet count, PDGF-BB:TNF-α log-ratio) from an inflammatory dimension (log(TNF-α), log(IL-1β)). Overall, hemocomponent composition emerged as the primary determinant of mediator organization, supporting the interpretation of PRG as a structured, biomaterial defined by coordinated mediator networks.

## 1. Introduction

Platelet-rich gel (PRG) is a biobased biomaterial generated from a platelet-rich plasma (PRP) precursor obtained through controlled processing of autologous blood. Upon activation, this liquid precursor is transformed into a gel, giving rise to a fibrin-based matrix that entraps platelets, soluble mediators, and residual cellular elements. Within commonly used classification frameworks, PRP-derived products are defined primarily by their white blood cell (WBC) content and the structural characteristics of the fibrin network formed during gelation. In this context, PRG derived from a WBC-poor PRP precursor is classified as a pure platelet-rich gel (P-PRG), characterized by limited WBC carryover and the formation of a low-density fibrin architecture following activation. These features distinguish PRG from fibrin-rich platelet products and confer distinct biological and structural properties that are central to its identity as a biomaterial [1,2,3].

As a fibrin-based hydrogel derived from autologous blood, PRG fulfills key criteria of a biobased gel. It originates from renewable biological resources, is biodegradable, exhibits low toxicity, and forms a hydrated three-dimensional fibrin network capable of entrapping cells and bioactive molecules [4,5]. While classical gel research often emphasizes physicochemical or rheological characterization, platelet-based gels also possess an embedded biological dimension arising from coordinated interactions among platelets, residual leukocytes, and soluble mediators within the matrix [3]. The present study focuses specifically on this biological organizational aspect of a fibrin-based biobased gel, rather than on its mechanical or viscoelastic properties, in order to better understand its mediator architecture in regenerative contexts.

Despite their growing application, the biological characterization of PRG-based biomaterials has largely relied on univariate assessments of isolated mediators, most commonly individual growth factors (GF) or inflammatory cytokines [6,7,8,9]. However, such an approach may fragment the biological context and fail to capture the integrated structure of these systems. In PRG matrices, multiple mediators and cellular components coexist and interact within the same environment. As a result, comparisons among PRP-derived hemocomponents are often based on partial descriptors that may overlook higher-order biological organization [10,11,12].

Within PRG-based biomaterials, biological activity arises from the combined presence of platelet- and leukocyte derived GF, inflammatory and regulatory cytokines, and residual cellular components, each contributing distinct but interconnected functions [1,13]. GF such as platelet-derived growth factor-BB (PDGF-BB) and transforming growth factor beta-1 (TGF-β1) play central roles in cell proliferation, matrix remodeling, and tissue repair, and are widely regarded as key trophic mediators associated with platelet activation. In parallel, cytokines including tumor necrosis factor alpha (TNF-α), interleukin-1 beta (IL-1β), interleukin-6 (IL-6), and interleukin-10 (IL-10) participate in the regulation of inflammatory signaling, immune modulation, and resolution processes, contributing to the dynamic balance between pro-inflammatory and regulatory pathways within the gel matrix [7,14,15].

Importantly, these mediators do not act in isolation within PRG but coexist within the same fibrin-based environment, where their relative abundance and interactions may shape the overall biological identity of the biomaterial. The simultaneous presence of trophic and regulatory signals suggests that PRG function is better understood in terms of mediator balance and coordinated behavior rather than absolute concentrations of individual molecules [16]. For this reason, the inclusion of GF, cytokines, and hematological variables in the present study was intended to capture complementary dimensions of PRG biology, enabling assessment of how platelet-derived trophic signals and inflammatory regulators collectively contribute to the organization of this living biomaterial [17].

From a biomaterials perspective, PRG and its related hemocomponents should be regarded as complex biological systems rather than as carriers of single bioactive molecules [15,16]. Multivariate analytical approaches offer a framework to address this complexity by integrating multiple variables simultaneously and identifying latent structures that reflect coordinated biological behavior. Such approaches are particularly relevant for biobased gels, whose properties emerge from the collective interaction of molecular and cellular constituents rather than from isolated components [18].

PRP and PRG are widely used in regenerative medicine as autologous biological therapeutics rather than merely analytical constructs. In both human and veterinary medicine, PRP/PRG preparations are applied in the management of musculoskeletal injuries (i.e., tendon and ligament lesions, osteoarthritis), soft tissue repair, and adjunctive wound healing protocols, where their proposed benefit derives from the coordinated release of growth factors and immunomodulatory mediators within a fibrin-based scaffold [7,19,20]. In clinical veterinary practice, dogs frequently receive non-steroidal anti-inflammatory drugs (NSAIDs) during peri-procedural management of such conditions [21]. Therefore, understanding whether short-term NSAID exposure perturbs the biological organization of platelet-derived gels is clinically relevant, even when pharmacological modulation is not the primary endpoint of investigation.

Canine models provide a valuable translational context for the study of platelet-based biomaterials, given their clinical relevance and frequent use in regenerative applications such as osteoarthritis, soft tissue musculoskeletal disorders, and wound repair [8,22,23,24,25]. In routine veterinary practice, PRG are often prepared and applied under real-world conditions that include concurrent pharmacological treatments. Among these, non-steroidal anti-inflammatory drugs are commonly administered and may influence inflammatory and trophic mediators [21,26]. However, the extent to which such exposure alters the global biological organization of PRG-based biomaterials remains unclear.

Therefore, the aim of this study was to explore the multivariate biological organization of PRG and related hemocomponents as fibrin-based biobased gels in a canine model under single NSAID exposure. Rather than focusing on isolated mediator responses, this work examines coordinated mediator patterns within the gel matrix. Using multivariate statistical analyses, we evaluated whether the observed organizational patterns are primarily associated with hemocomponent composition or influenced by short-term pharmacological context. In doing so, this study provides an exploratory multivariate perspective for the biological characterization of platelet-based gels used in regenerative medicine.

## 2. Results and Discussion

### 2.1. Cellular and Multivariate Organization of Platelet-Based Hemocomponents

PRG was generated from a PRP precursor with moderate platelet enrichment (~1.6-fold relative to whole blood) and marked WBC reduction (~8–9-fold decrease) (Table 1). These results indicate limited leukocyte carryover into the gel matrix (Table 1). In contrast, plasma (negative control) showed profound depletion of both platelets (~9–10-fold reduction) and WBC (~24-fold reduction), clearly distinguishing it from platelet-derived hemocomponents.

Cellular profiles remained stable across time (0 and 6 h) and NSAID exposure (carprofen (CAR) or firocoxib (FIR)), confirming that all experimental conditions were based on a comparable cellular substrate. Therefore, subsequent differences in GF and cytokine profiles are unlikely to reflect variation in initial cellular input.

Based on platelet enrichment combined with WBC reduction, both the PRP precursor and the resulting PRG can be classified as pure platelet concentrates (P-PRP/P-PRG) according to the Dohan Ehrenfest classification [3,27]. This distinction is relevant, as mediator patterns in P-PRG are expected to be driven primarily by platelet-derived factors and matrix interactions rather than by leukocyte-dominated inflammatory processes. Accordingly, the present findings should not be extrapolated to leukocyte-rich PRP formulations.

This perspective underscores the importance of explicitly defining and classifying PRG when reporting biological or functional analyses. Without such clarification, comparisons across studies may inadvertently group distinct platelet-derived preparations under the same terminology, potentially complicating interpretation of biological differences [1].

Descriptive mediator profiling revealed clear differences among hemocomponents. Plasma consistently exhibited the lowest GF concentrations, consistent with its marked cellular depletion. PDGF-BB values were lower in PRG compared with PRP and chemical-induced platelet lysate (CIPL), suggesting redistribution or matrix-associated retention within the gel structure.

**Figure 1:**
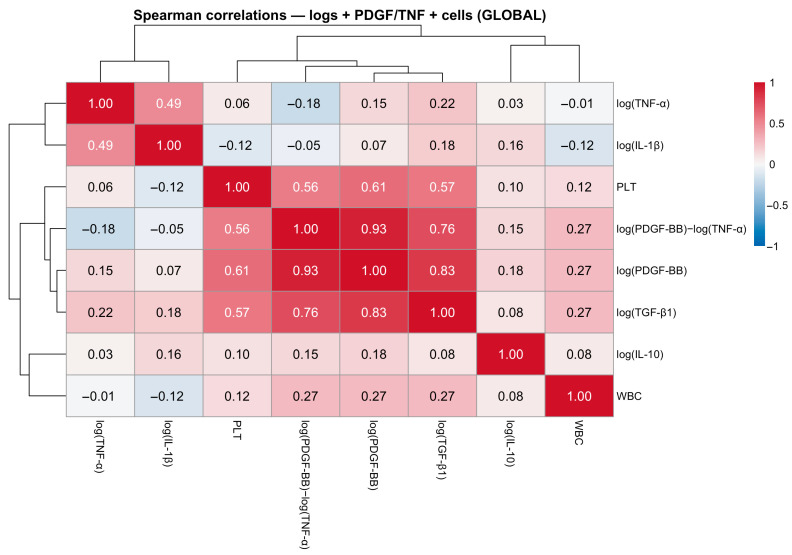
Global correlation structure among mediators and cellular variables in platelet-based hemocomponents. Heatmap of Spearman correlation coefficients (ρ), calculated using log-transformed growth factors (GF) and cytokines, the PDGF-BB/TNF-α log-ratio (log(PDGF-BB) − log(TNF-α)), and hematological variables (platelet and white blood cell counts). Correlation coefficients are displayed within each cell. Color intensity represents the direction and magnitude of the association. The matrix reveals a platelet-associated trophic block linking PLT, log(PDGF-BB), log(TGF-β1), and the PDGF-BB/TNF-α log-ratio, as well as a partially independent inflammatory block characterized by log(TNF-α) and log(IL-1β). White blood cell count (WBC) and log(IL-10) show weaker associations with both clusters. Acronyms as in Table 1.

Global Spearman correlation analysis revealed a structured pattern of associations (Figure 1). A platelet-associated trophic block linked log(PDGF-BB), log(TGF-β1), platelet count, and the PDGF-BB:TNF-α log-ratio. Inflammatory mediators (log(TNF-α), log(IL-1β)) formed a partially independent cluster. These findings indicate coordinated but partially segregated mediator patterns within platelet-based hemocomponents.

PRG differs from acellular platelet lysates by retaining cellular elements within a fibrin matrix. Even with reduced WBC content, residual leukocytes may contribute to cytokine balance. Thus, mediator organization in PRG likely reflects interactions among platelets, residual leukocytes, and the matrix structure rather than isolated protein concentrations alone.

This distinction may have implications for the interpretation and comparative evaluation of platelet-based biomaterials. Approaches that prioritize protein yield or compositional simplicity may enhance reproducibility but may not fully capture differences related to matrix-associated cellular context that distinguish PRG from acellular derivatives [27]. Accordingly, mediator balance in PRG should be interpreted in light of its combined cellular and fibrin matrix components rather than solely on the basis of isolated protein concentrations.

### 2.2. Linear and Mixed-Effects Modeling of the PDGF-BB/TNF-α Ratio

To quantify the relative balance between trophic and inflammatory signaling, the PDGF-BB:TNF-α log-ratio was modeled as a function of hemocomponent type, time (0 vs. 6 h), NSAID exposure (CAR vs. FIR), and WBC, including dog identity as a random intercept to account for repeated measures. Fixed-effect estimates and inferential statistics derived from the linear mixed-effects model are reported in Table 2.

**Figure 2:**
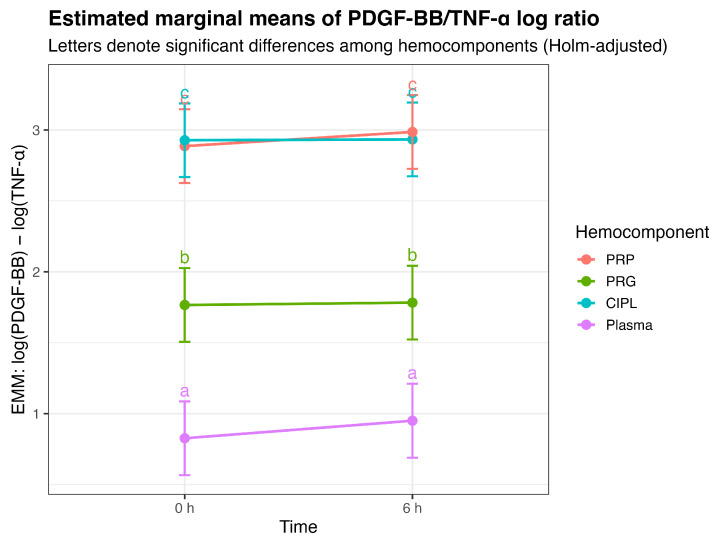
Estimated marginal means (±95% CI) of the PDGF-BB:TNF-α log-ratio for each hemocomponent at 0 and 6 h. Different letters denote Holm-adjusted pairwise differences among hemocomponents (mixed-effects model). No time or hemocomponent-by-time effects were supported, indicating temporal stability of the ratio under single-dose NSAID exposure. Acronyms as in Table 1.

Estimated marginal means showed high stability of the PDGF-BB:TNF-α ratio across time within each hemocomponent (i.e., PRP: 2.89 at 0 h vs. 2.99 at 6 h; PRG: 1.77 vs. 1.78). The clear separation among hemocomponents and the absence of relevant temporal effects are visually summarized in Figure 2, supporting the interpretation of the PDGF-BB:TNF-α ratio as a structural descriptor of hemocomponent identity rather than as a time- or NSAID-sensitive endpoint under single-dose exposure.

Sensitivity analysis using a Gaussian (identity) generalized linear mixed model yielded coefficient estimates nearly identical in magnitude, direction, and statistical inference to those obtained with the primary LMM (Table S1), supporting robustness of the results to distributional assumptions.

In clinical practice, TNF-α is often regarded as a pro-inflammatory cytokine that may limit regenerative strategies [28]. However, TNF-α signaling occurs through two receptors, TNFR1 and TNFR2, which activate partially divergent downstream pathways [29]. TNFR1 is typically associated with inflammatory and apoptotic responses, whereas TNFR2 has been implicated in immune modulation and tissue repair, highlighting the context-dependent nature of TNF-α signaling [30].

Cytokines within platelet-based preparations operate within interconnected signaling environments rather than as isolated mediators [31]. Previous in vitro studies have shown that PRG supernatants can downregulate NF-κB expression and related catabolic genes [32,33]. Although receptor-specific signaling was not evaluated in the present study, these prior observations illustrate that TNF-α activity cannot be interpreted solely on the basis of concentration. Any mechanistic interpretation in this context remains hypothesis-generating and requires direct experimental confirmation.

In pure platelet concentrates, leukocyte content is reduced but not absent. Residual leukocyte-derived TNF-α may therefore contribute to local signaling balance in a context-dependent manner [34]. Within this framework, the PDGF-BB:TNF-α log-ratio can be viewed as a quantitative representation of relative mediator balance rather than as a direct indicator of inflammatory status.

Importantly, the magnitude of the hemocomponent effects indicates shifts in mediator balance associated with preparation type. The negative coefficient observed for PRG (−1.12) corresponds to a reduction in the PDGF-BB:TNF-α ratio relative to PRP, while plasma exhibited a larger decrease (−2.06). These differences are consistent with compositional characteristics of each hemocomponent rather than transient temporal modulation.

Neither post-exposure time nor NSAID type significantly influenced the ratio, and WBC count was not retained as a relevant predictor. The absence of a detectable WBC effect suggests that variation in the PDGF-BB:TNF-α balance was more closely associated with hemocomponent category than with leukocyte quantity alone. This pattern is consistent with the interpretation that mediator relationships in PRG reflect coordinated interactions within the hemocomponent matrix rather than simple dependence on residual cell counts [35].

### 2.3. Principal Component Analysis (PCA)

**Figure 3:**
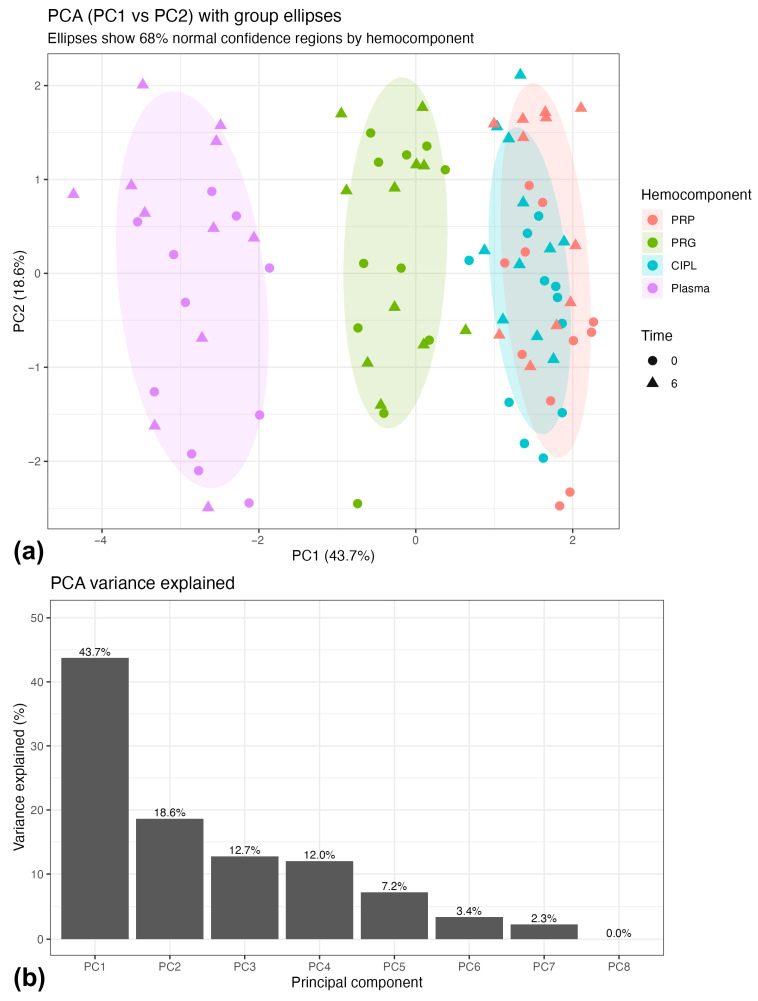
(**a**) PCA score plot showing the distribution of samples along PC1 (43.7%) and PC2 (18.6%), with 68% normal confidence ellipses by hemocomponent. Separation patterns were primarily associated with hemocomponent composition, while overlap between time points (0 and 6 h) indicates minimal temporal effects at the multivariate level. (**b**) Scree plot illustrating the proportion of variance explained by each principal component; the first two components accounted for 61.3% of the total variance. Subsequent components (PC3–PC8) explained progressively smaller proportions of variance and did not reveal additional interpretable structure; therefore, interpretation focused on PC1 and PC2. Acronyms as in Table 1.

PCA was applied to the integrated dataset, including log-transformed mediators, the PDGF-BB:TNF-α log-ratio, and hematological variables, using centered and scaled data. The first two principal components explained 61.3% of total variance (PC1 = 43.7%, PC2 = 18.6%) (Figure 3a). PC1 showed high positive loadings for log(PDGF-BB) (0.52), the PDGF-BB:TNF-α log-ratio (0.51), log(TGF-β1) (0.48), and platelet count (0.47), consistent with a platelet-associated trophic dimension (Figure 3b).

PC2 was primarily defined by log(IL-1β) (−0.68) and log(TNF-α) (−0.66), indicating a largely independent inflammatory dimension. Cluster bootstrap resampling supported the stability of these loadings despite the limited number of independent animals. Variables contributing to PC1 and PC2 retained consistent sign and magnitude across bootstrap replicates, with confidence intervals not crossing zero, supporting structural stability of the first two components.

**Figure 4:**
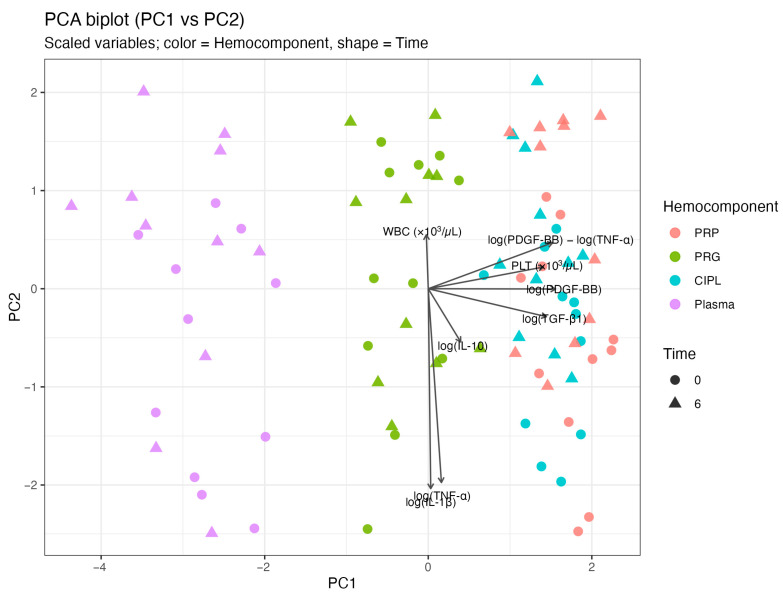
PCA biplot of PC1 versus PC2 based on centered and scaled variables. PC1 was mainly defined by platelet-associated variables [log(PDGF-BB), log(TGF-β1), platelet count (PLT), and the PDGF-BB:TNF-α log-ratio], representing a dominant platelet/trophic axis. PC2 was driven primarily by inflammatory mediators [log(TNF-α) and log(IL-1β)], defining an orthogonal inflammatory axis. Sample clustering by hemocomponent was evident, whereas grouping by time or NSAID exposure was not observed. Acronyms as in Figure 1 and Table 1.

Projection of samples in the PC space showed separation primarily according to hemocomponent type, whereas clustering by time or NSAID exposure was minimal at the multivariate level (Figure 4). The positive loading of the PDGF-BB:TNF-α log-ratio on PC1 indicates that the ratio aligns with platelet-associated variables, in agreement with the mixed-effects modeling results.

It is important to clarify that the present findings should be interpreted in light of our previously published univariate analysis of this same experimental dataset [25]. In that primary study, specific mediator-level effects were identified, including interaction effects for TGF-β1 and selected cytokines, suggesting that a single therapeutic dose of carprofen or firocoxib may transiently modulate the release dynamics of certain mediators under short-term conditions. Therefore, the current results do not imply a complete absence of NSAID influence. Rather, they indicate that, when mediator behavior is examined from a multivariate and integrative perspective, short-term single-dose exposure does not appear to induce a major reorganization of the overall mediator network structure across hemocomponents.

This distinction is central to the interpretation of the pharmacological component of the study. The design (single dose, 6 h window, no pharmacodynamic confirmation, or platelet function testing) does not allow conclusions regarding chronic administration, cumulative COX-2 inhibition, or long-term regenerative performance. Accordingly, the present multivariate findings should not be interpreted as evidence that NSAIDs are biologically neutral with respect to PRG, but rather that under acute, clinically realistic peri-procedural exposure, the global structural relationships among trophic and inflammatory mediators remain relatively stable. More comprehensive pharmacological studies incorporating repeated dosing, longer observation windows, biomarker confirmation of COX inhibition, and functional platelet assays would be required to determine whether sustained NSAID exposure meaningfully alters PRG biological activity.

The orthogonality between PC1 and PC2 suggests that trophic and inflammatory signals contributed to partially independent dimensions rather than representing a simple linear opposition. Consistent patterns across PCA and LMM analyses indicate that hemocomponent category was more strongly associated with mediator structure than short-term NSAID exposure.

From an analytical standpoint, these findings illustrate the utility of multivariate approaches for summarizing coordinated mediator patterns in platelet-based preparations [18].

The emergence of distinct organizational axes is consistent with systems-level principles in which biological behavior arises from coordinated network interactions rather than from single-variable effects [35]. The observed covariance structure is compatible with models in which biological behavior reflects interacting mediator networks rather than isolated variables.

### 2.4. Heatmap and Hierarchical Clustering Analyses

**Figure 5:**
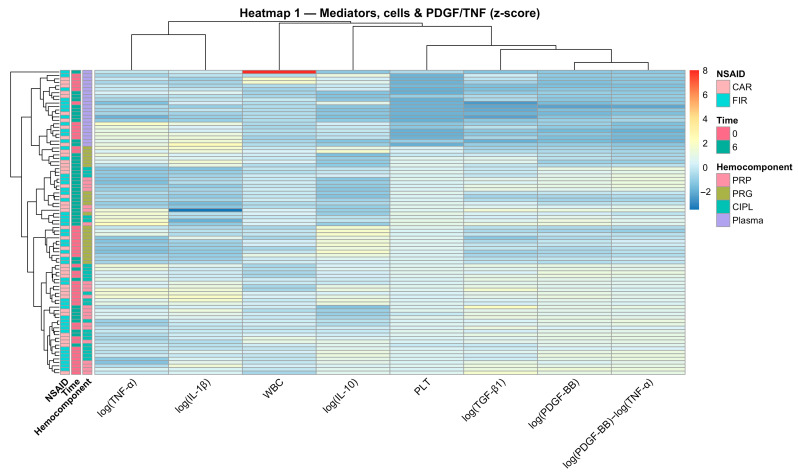
Heatmap of mediators, cellular variables, and PDGF-BB/TNF-α ratio at the sample level. Heatmap-based visualization of log-transformed soluble mediators, cell counts, and the PDGF-BB:TNF-α log-ratio using z-score normalization and hierarchical clustering. Samples are shown in rows and variables in columns. Platelet-associated features cluster separately from inflammatory mediators, reproducing the block structure observed in correlation analyses and PCA. Side annotations indicate hemocomponent type, Time (0 or 6 h), and NSAID exposure (CAR or FIR). Acronyms as in Figure 1 and Table 1.

Heatmap-based visualization combined with hierarchical clustering provided a complementary, sample-level perspective on the multivariate organization identified by PCA and mixed-effects modeling [18,36]. Using z-score normalization, the integrated heatmap of soluble mediators, cellular variables, and the PDGF-BB:TNF-α log-ratio reproduced the platelet-associated and inflammatory block structure observed in correlation analyses and reflected in the orthogonal PC1 and PC2 axes (Figure 5). The co-clustering of log(PDGF-BB), log(TGF-β1), PLT, and the PDGF-BB:TNF-α log-ratio supports interpreting this ratio as aligned with the dominant platelet/trophic axis, rather than as an isolated derived metric.

Importantly, sample clustering was driven primarily by hemocomponent identity, with minimal separation attributable to time or NSAID exposure. This visual pattern is consistent with the linear mixed-effects results, which demonstrated significant hemocomponent effects but no relevant temporal, pharmacological, or WBC-dependent modulation of the PDGF-BB:TNF-α ratio. Thus, heatmap clustering indicates that mediator balance remained stable within each preparation under the evaluated conditions.

**Figure 6:**
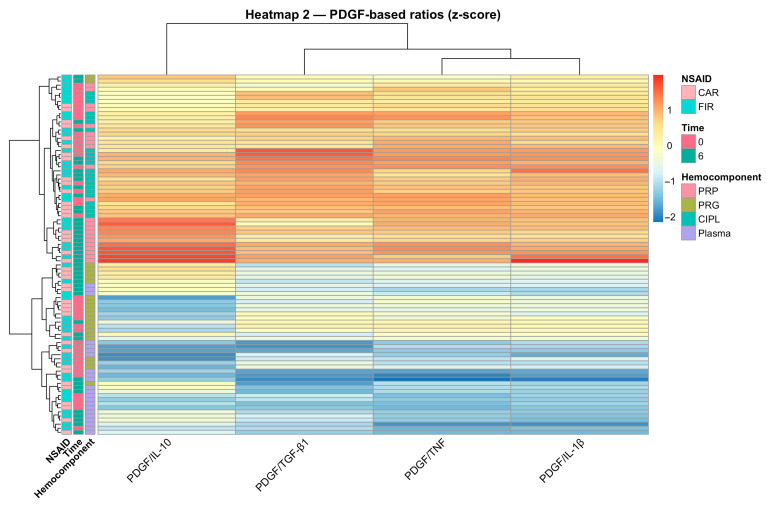
Heatmap of PDGF-based ratios across samples. Heatmap of z-score-normalized PDGF-based ratios with hierarchical clustering at the sample level. The PDGF-BB:TNF-α log-ratio shows the most consistent and discriminative pattern across hemocomponent preparations, whereas alternative PDGF-based ratios display partially overlapping and less distinct profiles. Side annotations indicate hemocomponent type, Time (0 or 6 h), and NSAID exposure (CAR or FIR). Acronyms as in Figure 1 and Table 1.

The second heatmap, focused on PDGF-BB-based ratios, showed that the PDGF-BB:TNF-α log-ratio provided the most consistent contrast across hemocomponents (Figure 6). Alternative ratios displayed partial redundancy and weaker separation patterns, suggesting that not all GF–cytokine combinations contributed equally to distinguishing preparation types. The recurrent alignment of the PDGF-BB:TNF-α ratio across PCA, LMM, and clustering analyses supports its interpretation as a compositional descriptor within platelet-based systems.

**Figure 7:**
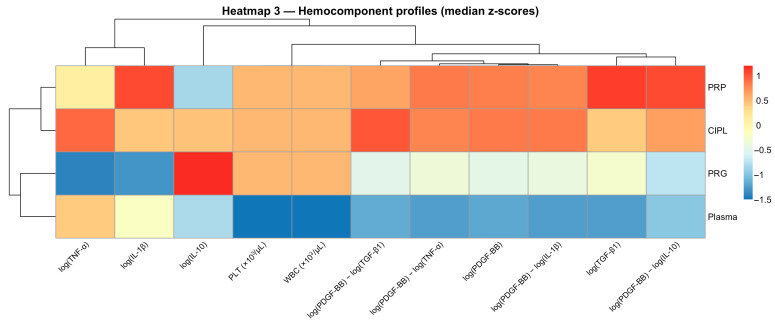
Hemocomponent-level biological profiles based on median z-scores. Heatmap summarizing hemocomponent-specific biological profiles using median z-scores for mediators, cellular variables, and PDGF-based ratios. Each row represents a hemocomponent and each column a scaled variable. Distinct multivariate “hemocomponent-specific multivariate patterns” are evident for each preparation, highlighting systematic differences driven by hemocomponent composition rather than by Time or NSAID exposure. PDGF-BB:TNF-α log-ratio, calculated as log(PDGF-BB) − log(TNF-α); PDGF-BB:TGF-β1 log-ratio, calculated as log(PDGF-BB) − log(TGF-β1). Acronyms as in Figure 1 and Table 1.

Finally, hemocomponent-level heatmaps based on median z-scores summarized distinct “hemocomponent-specific multivariate patterns” for each preparation (Figure 7). These profiles integrate trophic signals, inflammatory mediators, and cellular variables into coherent configurations. Together, they reflect intrinsic compositional characteristics. The consistency of these patterns across analytical approaches (mixed modeling, dimensional reduction, and hierarchical clustering) supports the interpretation that hemocomponent category was the primary factor associated with mediator organization under the evaluated conditions. From an analytical perspective, these integrative visualization approaches allow high-dimensional mediator interactions to be summarized into structured profiles [10,11,12,35]. When considered together with PCA and mixed-effects modeling, the results consistently indicate that hemocomponent composition was more strongly associated with mediator configuration than time or single-dose NSAID exposure.

The convergence observed across supervised and unsupervised analytical strategies indicates internal structural consistency within the dataset [18,36]. From a systems-oriented analytical perspective, this coherence suggests that mediator relationships within platelet-based gels are organized in reproducible multivariate configurations rather than occurring as independent fluctuations of isolated molecules [12,35]. Accordingly, PRG may be more appropriately described in terms of coordinated, multivariate mediator organization within this experimental context, rather than as a random cytokine mixture.

From a comparative standpoint, such profiles may facilitate classification and analytical comparison of platelet-based preparations without implying direct functional equivalence. This underscores the value of multivariate visualization tools for summarizing biological complexity in biobased gels.

Taken together, descriptive analyses, mixed-effects modeling, PCA, and hierarchical clustering consistently indicated that hemocomponent composition was more strongly associated with mediator organization than time or single-dose NSAID exposure in this dataset. The PDGF-BB:TNF-α log-ratio aligned with the dominant platelet/trophic axis and remained stable across experimental conditions, supporting its interpretation as a compositional descriptor rather than a transient pharmacological response.

The orthogonal separation of trophic- and inflammatory-associated dimensions further indicates that these mediator groups contributed to partially independent multivariate patterns within the analyzed space, rather than representing a simple linear opposition.

This study extends PRG characterization beyond univariate mediator quantification by applying an integrated multivariate analytical framework [8,22,24,25]. By combining linear mixed-effects modeling with dimensional reduction and clustering approaches [18,36], the analyses revealed reproducible multivariate structure across methods. The identification of the PDGF-BB:TNF-α log-ratio as a stable descriptor of trophic–inflammatory balance provides a quantitative metric for examining mediator relationships within biologically active fibrin-based gels. Within the analytical scope of this study, these findings are consistent with systems-oriented models of inflammation, in which mediator behavior is interpreted in terms of coordinated interactions rather than isolated molecular concentrations [10,11,12,35].

An additional methodological strength of this study is the use of cluster bootstrap resampling at the subject level to assess loading stability of the principal components. This approach preserves the within-dog dependency structure inherent to the crossover design and provides empirical support for the stability of the trophic- and inflammatory-associated axes within this dataset, while acknowledging the limited number of independent animals [18,37,38].

Several limitations should be acknowledged. The cohort size was limited (n = 6), and although the crossover design improves control of inter-individual variability, the individual animal remained the effective experimental unit. Accordingly, the identified multivariate organization should be interpreted within an internally consistent but restricted sample, and broader generalization requires independent confirmation. The experimental design prioritized statistical efficiency while minimizing animal use, in accordance with reduction principles in biomedical research.

Unsupervised techniques such as PCA and hierarchical clustering are sensitive to sample size and to the covariance structure of the variables. To mitigate this, loading stability was evaluated using cluster bootstrap resampling at the subject level, preserving within-dog dependency and supporting the stability of the trophic- and inflammatory-associated axes within this dataset [18,37,38]. Nonetheless, replication in larger independent cohorts will be necessary to confirm reproducibility.

Because the PDGF-BB:TNF-α ratio is mathematically derived from mediators included in the multivariate matrix, partial alignment between the ratio and principal component axes reflects shared covariance structure. This alignment was interpreted as evidence of coordinated multivariate structure within the dataset rather than as independent biological validation, and should be understood within that analytical framework [12,31,35].

Potential collinearity among inflammatory and growth mediators is biologically plausible and may influence multivariate loadings and clustering topology [12,31,35]. Receptor-level signaling pathways, including TNFR1/TNFR2 activation and downstream NF-κB signaling, were not directly assessed; therefore, mechanistic interpretations remain hypothesis-generating. In addition, the mediator panel was targeted rather than comprehensive, and the pharmacological component was limited to short-term evaluation following a single dose of COX-2 selective NSAIDs, restricting extrapolation to chronic or repeated dosing scenarios.

It is important to emphasize that the present study does not aim to characterize the rheological, ultrastructural, or mechanical properties of the fibrin matrix. Rather, it addresses the biological mediator organization embedded within a fibrin-based biobased gel. These dimensions are complementary and not mutually exclusive, and future studies integrating physicochemical and biological characterization would provide a more comprehensive understanding of platelet-derived hydrogels.

Finally, the present work represents a secondary integrative analysis of an existing experimental dataset [25]. Although the analytical objective was distinct and extended through multivariate modeling and resampling procedures, prospective validation in independent cohorts and mechanistic studies will be necessary to confirm broader applicability.

## 3. Conclusions

This exploratory study suggests that PRG and related hemocomponents exhibit a reproducible multivariate biological organization within the evaluated cohort, primarily associated with hemocomponent composition rather than short-term post-preparation time or single-dose NSAID exposure. Across mixed-effects modeling, principal component analysis, and hierarchical clustering, the PDGF-BB:TNF-α log-ratio consistently aligned with the dominant platelet-associated axis and remained stable under the limited pharmacological conditions tested, supporting its potential utility as an internal descriptor of mediator balance within the evaluated experimental framework.

The observed orthogonal separation of trophic and inflammatory dimensions and the persistence of hemocomponent-specific multivariate patterns indicate that PRG may be more appropriately described in terms of coordinated mediator organization rather than as a simple aggregate of independent cytokine concentrations. By integrating statistical modeling with biologically informed interpretation, this work provides an exploratory multivariate perspective for characterizing the biological dimension of fibrin-based biobased gels used in regenerative applications.

## 4. Materials and Methods

This prospective, randomized, controlled crossover study was reviewed and approved by the Animal Experimentation Committee of Universidad de Caldas (Manizales, Colombia; Project code PRY34-2023; approval date: 19 June 2023). Dog owners were informed about the nature and procedures of the study and provided written informed consent prior to participation.

### 4.1. Study Design and Data Source

The present study represents a secondary analysis of a previously generated controlled experimental dataset of canine platelet-based gels [25]. The original experimental protocol included the preparation of PRG and related hemocomponents derived from canine whole blood under standardized laboratory conditions. The design incorporated predefined sampling time points (0 and 6 h) and single-dose exposure to two COX-2–selective NSAIDs commonly used in canine practice, CAR and FIR [21,26].

The study followed a randomized two-period crossover design in which each dog received carprofen and firocoxib in separate experimental phases, with an appropriate washout interval (three weeks) between treatments as described in the original study [25]. Each NSAID was administered once per experimental phase at standard therapeutic dosage.

While the primary study focused on univariate drug- and time-dependent biological effects, the present re-analysis was designed with a distinct objective: to examine the multivariate biological organization of PRG and related hemocomponents.

The experimental unit was the individual dog (n = 6) within a randomized crossover design. The cohort included adult dogs of both sexes, as detailed in the original publication [25]. Multiple hemocomponents were obtained from each animal at baseline and 6 h following NSAIDs administration.

### 4.2. Experimental Model and Hemocomponent Preparation

Blood samples were obtained from six adult dogs under controlled experimental conditions using a within-subject design. At baseline (time 0 h), autologous whole blood was collected from each animal and processed to generate the panel of hemocomponents evaluated in this study, including plasma, PRG derived from a PRP precursor, and CIPL. This design allowed direct comparison of biologically related hemocomponents generated from the same individual while minimizing inter-individual variability.

PRP was prepared using a standardized centrifugation-based protocol designed to yield a leukocyte-poor platelet concentrate [24]. PRG was generated by activating PRP with calcium gluconate, inducing fibrin polymerization and gel formation. Following activation, the gel-derived fraction was collected for subsequent analyses. Plasma samples obtained from the same blood draws were included as comparative hemocomponents representing matrices with low platelet and cellular content.

CIPL (positive control for GF enrichment) was generated from PRP by inducing complete platelet lysis through exposure to a non-ionic detergent, resulting in a cell-free lysate enriched in platelet-derived mediators. This hemocomponent was included as an acellular platelet-derived reference to contrast lysate-based preparations with living platelet-based gels retaining intact cellular elements.

Immediately after baseline sampling and hemocomponent preparation, dogs received a single therapeutic dose of one of two NSAIDs (either CAR or FIR) administered according to standard veterinary practice. Six hours after drug administration, a second blood sample was collected from each animal, and the same panel of hemocomponents was prepared again following identical procedures. This approach allowed paired evaluation of hemocomponents generated before and after NSAID exposure under otherwise comparable conditions.

The single-dose exposure model was selected to evaluate short-term peri-procedural pharmacological effects rather than chronic NSAID administration. The design was not intended to replicate long-term pharmacodynamic effects.

All hemocomponents were produced and processed under standardized conditions within each animal to ensure direct comparability across time points and experimental conditions. In experiments involving repeated NSAID exposure, experimental sessions were separated by an appropriate washout period to minimize potential carryover effects. Detailed information regarding animal management, crossover allocation, NSAID administration, and washout duration is provided in the original experimental study [25].

### 4.3. Mediator Quantification

GF and cytokine concentrations were quantified by enzyme-linked immunosorbent assays (ELISA) performed in duplicate using DuoSet ELISA Development Kits (R&D Systems, Minneapolis, MN, USA), including Human PDGF-BB (DY220), Human TGF-β1 (DY240E), Canine IL-1β (DY3747), Canine TNF-α (DY1507), and Canine IL-10 (DY735). Although human ELISA kits were used for PDGF-BB and TGF-β1, these assays have been previously validated and widely applied in canine studies [24,25] due to the high interspecies homology of these proteins [39,40]. Absorbance was measured at 450 nm, and standard curves were constructed using the standards supplied with each kit in accordance with the manufacturers’ protocols.

Hematological variables, including platelet count and total WBC count, were obtained using an automated hematology analyzer (Celltac α MEK-6450. Nihon Kohden, Tokyo, Japan) and expressed as ×10^3^/µL. All laboratory measurements were performed under standardized conditions.

### 4.4. Statistical Analysis

All statistical analyses were performed using R software (version 4.3.2; R Foundation for Statistical Computing, Vienna, Austria) within a fully scripted and reproducible workflow [41]. Continuous mediator concentration data were log-transformed prior to analysis to reduce skewness and improve variance homogeneity.

Exploratory analyses included descriptive statistics and Spearman correlations among growth factors, cytokines, and hematological variables. A log-transformed PDGF-BB:TNF-α ratio was calculated to represent the relative balance between trophic and regulatory mediators and was used as a derived outcome variable.

Linear mixed-effects models (LMMs) were fitted to evaluate the association between the PDGF-BB:TNF-α ratio and hemocomponent type, post-treatment time (0 and 6 h), NSAID exposure (CAR or FIR), and selected hematological variables. Dog identity was included as a random intercept to account for within-subject correlation inherent to the crossover design. Models were estimated using restricted maximum likelihood (REML). Random-effect contribution was evaluated through variance components and singularity diagnostics, and simplified linear models were examined when appropriate to confirm stability of fixed-effect estimates.

Model assumptions were assessed using residual and Q–Q plots. Variance components, intraclass correlation coefficients (ICC), and marginal and conditional R^2^ values were calculated to quantify model structure [41,42].

To examine multivariate organization, PCA was applied to centered and scaled data including log-transformed mediators, the PDGF-BB:TNF-α ratio, and hematological variables [18]. Loading stability was assessed using cluster bootstrap resampling at the dog level (B = 2000), preserving within-subject dependency [43]. Principal components were aligned to the reference solution to prevent sign inversion, and mean loadings with 95% confidence intervals were calculated [18]. PCA was interpreted as an exploratory dimensionality-reduction method applied to summarize covariance structure in the dataset [20]. The mathematical derivation of the PDGF-BB:TNF-α ratio was considered when interpreting covariance structure [44].

Hierarchical clustering and heatmaps were used as complementary visualization tools. Z-score normalization was applied at the variable level for both sample-level and hemocomponent-level summaries [36].

Data manipulation and visualization were performed using tidyverse packages (including dplyr and ggplot2). Models were estimated using restricted maximum likelihood (REML) as implemented in the lme4 package. Estimated marginal means were obtained using emmeans. Multivariate analyses were conducted using base R functions, and heatmaps were generated using pheatmap.

A two-sided significance level of *p* < 0.05 was adopted for exploratory univariate analyses; however, interpretation focused primarily on multivariate structure and consistency of biological patterns rather than on isolated *p*-values.

## Figures and Tables

**Table 1:** Descriptive hematological and mediator profiles of platelet-rich gel and related hemocomponents.

Hemocomponent	PLT × 10^3^/µL	WBC × 10^3^/µL	PDGF-BB (pg/mL)	TGF-β1 (pg/mL)	TNF-α (pg/mL)	IL-1β (pg/mL)	IL-10 (pg/mL)
PRP	305.50 (300.00–319.75)	0.90 (0.80–1.08)	630.05 (554.22–707.03)	17,016.54 (15,573.31–18,114.58)	32.20 (27.48–36.94)	67.69 (57.61–75.19)	30.11 (22.27–69.96)
PRG	305.50 (300.00–319.75)	0.90 (0.80–1.08)	180.29 (157.33–233.16)	10,047.21 (8931.56–10,796.96)	29.88 (26.85–37.94)	56.72 (53.50–69.21)	72.84 (22.56–116.20)
CIPL	305.50 (300.00–319.75)	0.90 (0.80–1.08)	658.04 (591.57–754.09)	12,916.35 (11,973.54–14,543.31)	33.51 (29.02–40.50)	64.59 (59.62–67.88)	52.17 (45.77–69.79)
Plasma	19.00 (11.25–27.00)	0.45 (0.23–0.50)	86.23 (71.74–96.98)	6937.96 (5582.27–8459.25)	32.68 (28.57–41.42)	61.56 (55.99–69.17)	31.38 (19.80–47.80)

Values are presented as median (interquartile range). Platelet and WBC counts are similar for PRP, PRG, and CIPL, as PRG and CIPL are generated from the same PRP precursor. PRP, platelet-rich plasma; PRG, platelet-rich gel; CIPL, chemically induced platelet lysate; PLT, platelet count; WBC, white blood cell count; PDGF-BB, platelet-derived growth factor-BB; TGF-β1, transforming growth factor beta-1; TNF-α, tumor necrosis factor alpha; IL-1β, interleukin-1 beta; IL-10, interleukin-10. Values are presented as median (interquartile range).

**Table 2:** Linear mixed-effects model for the PDGF-BB/TNF-α log-ratio.

Fixed Effect	Estimate	SE	95% CI	*t* Statistic	*p* Value
(Intercept, PRP at 0 h, CAR)	2.87	0.09	2.68 to 3.05	30.71	<0.001
Hemocomponent: PRG	−1.12	0.11	−1.34 to −0.90	−9.82	<0.001
Hemocomponent: CIPL	0.04	0.11	−0.18 to 0.27	0.36	0.72
Hemocomponent: Plasma	−2.06	0.11	−2.28 to −1.84	−18.05	<0.001
Time (6 h)	0.10	0.11	−0.12 to 0.32	0.88	0.38
NSAID (FIR)	0.01	0.06	−0.10 to 0.12	0.17	0.86
WBC	0.02	0.03	−0.04 to 0.08	0.63	0.53
PRG × Time	−0.08	0.16	−0.40 to 0.23	−0.52	0.61
CIPL × Time	−0.09	0.16	−0.41 to 0.22	−0.58	0.56
Plasma × Time	0.02	0.16	−0.29 to 0.34	0.15	0.88

PRP at 0 h under carprofen (CAR) exposure was used as the reference category. Dog identity was included as a random intercept. Confidence intervals (CI) correspond to Wald 95% intervals. Reported statistics correspond to t values from the linear mixed-effects model. The random intercept variance attributable to dog identity was 0.0041, with a residual variance of 0.0715, resulting in an intraclass correlation coefficient (ICC) of 0.055. Thus, approximately 5.5% of the total variance in the PDGF-BB:TNF-α ratio was attributable to between-dog variability, indicating that most variation was explained at the hemocomponent level rather than by individual differences. FIR, firocoxib; NSAID, non-steroidal anti-inflammatory drug. SE, Standard error. Other acronyms as in Table 1.

## Data Availability

The raw data supporting the conclusions of this article will be made available by the authors on request.

## Supplementary Materials

The following supporting information can be downloaded at: https://www.mdpi.com/article/10.3390/gels12030246/s1, Table S1. Sensitivity analysis using Gaussian (identity) generalized linear mixed model for the PDGF-BB:TNF-α log-ratio.

## Abbreviations

The following abbreviations are used in this manuscript:

PRP	Platelet-rich plasma
PRG	Platelet-rich gel
CIPL	Chemically induced platelet lysate
PLT	Platelet count
WBC	White blood cell count
PDGF-BB	Platelet-derived growth factor-BB
TGF-β1	Transforming growth factor beta-1
TNF-α	Tumor necrosis factor alpha
IL-1β	Interleukin-1 beta
IL-6	Interleukin-6
IL-10	Interleukin-10
NSAID	Non-steroidal anti-inflammatory drug
CAR	Carprofen
FIR	Firocoxib
COX-2	Cyclooxygenase-2
PCA	Principal component analysis
PC	Principal component
LMM	Linear mixed-effects model
GLMM	Generalized linear mixed-effects model
REML	Restricted maximum likelihood
CI	Confidence interval
ELISA	Enzyme-linked immunosorbent assay
NFκB	Nuclear factor kappa B
TNFR1	Tumor necrosis factor receptor 1
TNFR2	Tumor necrosis factor receptor 2
