# Supplementary material for: Exploratory Multivariate Analysis of Mediator Organization in Canine Platelet-Rich Gel Under NSAID Exposure
Source: Gels. 2026 Mar 14;12(3):246. doi: 10.3390/gels12030246 (PMC13025728; doi:10.3390/gels12030246)
Supplement: Supplementary file 1 [file gels-12-00246-s001.zip › gels-4173248-supplementary.pdf]

Table S1. Sensitivity analysis using Gaussian(identity) generalized linear mixed model for the PDGF-BB:TNF-α log-ratio.

Fixed Effect	Estimate	SE	z value	p value
(Intercept, PRP at 0 h, CAR)	2.865	0.094	30.34	<0.001
**Hemocomponent: PRG**	−1.120	0.114	−9.82	<0.001
**Hemocomponent: CIPL**	0.041	0.114	0.36	0.716
**Hemocomponent: Plasma**	−2.059	0.114	−18.05	<0.001
**Time (6 h)**	0.100	0.114	0.88	0.381
**NSAID (FIR)**	0.010	0.060	0.17	0.867
**WBC**	0.018	0.029	0.63	0.531
**PRG × Time**	−0.083	0.161	−0.52	0.606
**CIPL × Time**	−0.094	0.161	−0.58	0.560
**Plasma × Time**	0.024	0.162	0.15	0.882

Model specification: Gaussian family with identity link; random intercept for dog identity.
